# An anisotropic pore-network model to estimate the shale gas permeability

**DOI:** 10.1038/s41598-021-86829-4

**Published:** 2021-04-12

**Authors:** Di Zhang, Xinghao Zhang, Haohao Guo, Dantong Lin, Jay N. Meegoda, Liming Hu

**Affiliations:** 1 Department of Civil and Environmental Engineering, New Jersey Institute of Technology, Newark, NJ USA; 2 State Key Laboratory of Hydro-Science and Engineering, Department of Hydraulic Engineering, Tsinghua University, Beijing, China

**Keywords:** Petrology, Geodynamics, Structural geology, Solid Earth sciences, Energy science and technology

## Abstract

The permeability of shale is a significant and important design parameter for shale gas extraction. The shale gas permeability is usually obtained based on Darcy flow using standard laboratory permeability tests done on core samples, that do not account for different transport mechanisms at high pressures and anisotropic effects in shales due to nano-scale pore structure. In this study, the permeability of shale is predicted using a pore network model. The characteristics of pore structure can be described by specific parameters, including porosity, pore body and pore throat sizes and distributions and coordination numbers. The anisotropy was incorporated into the model using a coordination number ratio, and an algorithm that was developed for connections of pores in the shale formation. By predicting hydraulic connectivity and comparing it with several high-pressure permeability tests, the proposed three-dimensional pore network model was verified. Results show that the prediction from the anisotropic pore network model is closer to the test results than that based on the isotropic pore network model. The predicted permeability values from numerical simulation using anisotropic pore network model for four shales from Qaidam Basin, China are quite similar to those measured from laboratory tests. This study confirmed that the developed anisotropic three-dimensional pore network model could reasonably represent the natural gas flow in the actual shale formation so that it can be used as a prediction tool.

## Introduction

Extraction of natural gas from tight shale formations in the USA is one of the landmark events of the twenty-first century^1^. Horizontal drilling combined with hydraulic fracturing has allowed the extraction of large amounts of natural gas from low permeability tight shale formations that were previously considered impossible or uneconomical to exploit^2^. According to the US Energy Information Administration, as of 2012, shale gas in the United States accounts for more than 40% of the total domestic natural gas production and is expected to reach 50% by 2039^3^.

The permeability of shale is a crucial design parameter for gas extraction^4^. Shale is a heterogeneous and anisotropic ultra-fine rock with low pore connectivity, and extremely low intrinsic permeabilities of 10^–18^ to 10^–21^ (in m^2^)^5–7^. The permeability test of shales using traditional laboratory techniques may not provide realistic values. This has to do with the measurement of in-situ permeability of nanoscale pores at high pressures. Core samples with stress-free fractures and atmospheric moisture plus Darcy flow interpretation for different flow regimes at diffident applied pressures may result in substantial deviation from in-situ values. Hence it is important to understand the shale pore structure and develop a predictive tool for gas flow mechanisms using shale reservoir properties and shale pore structure.

At present, the research on pore-scale flow characteristics mainly focuses on typical geotechnical media such as sand and sandstone with millimeter or micrometer pores. As geotechnical engineering seepage research gradually expands to rock formations and even shales with micro-nano scale pores, the traditional flow analysis cannot reasonably explain the fluid flow characteristics at the micro-nano pore scale. The seepage characteristics of tight shale formations can be studied from a microscopic perspective using a pore network model^6^.

### Previous studies

A Pore network model can be used to investigate the micromechanics of seepage in porous media. Typical tight gas formations are usually porous media where nano-scale pores are randomly distributed within the shale matrix. Digital methods including focused-ion-beam scanning-electron-microscopy (FIB-SEM) and computed tomography (CT) are used to obtain two-dimensional slices of the pore structure of the shale samples, then three-dimensional pore reconstruction models can be built based on slices. Previous works have reported different methods of pore reconstruction^7–9^. Vega et al.^10^ studied the pore structure of Barnett, Eagle Ford, and Haynesville shale samples by using high X-ray contrast gas. Song et al.^11^ reports a local-effective-viscosity multi-relaxation-time lattice Boltzmann model to simulate the gas transport in shale. All previous research provide important information on shale structure and the gas transport.

Direct simulation using pore reconstruction models can be computationally demanding due to the complicated boundary conditions for flow and gas transport in the pore structure of shale. Alternatively, the equivalent pore network model is based on the statistical values of physical properties of rock and soil media to establish a simplified pore structure reflecting pore distributions and spatial connectivity. The equivalent pore network model was first proposed by Fatt^12^ in 1956. He proposed a two-dimensional network model based on capillary tubes, and combined the spherical pore accumulation model to account for the connectivity between pores, and proposed a two-dimensional regular grid network model. Nicholson and Petropoulos^13^ in 1971 established a three-dimensional equivalent pore network model and performed a quasi–static two-phase flow calculation. Since then, many scholars have studied the pore morphology of the pore network model. Dullien^14^ in 1979 simplified the connecting throats between pores into continuous capillary sections. Reeves and Celia^15^ in 1996 established a constant coordination number equivalent pore network model and further considered the linear change of pore throats. Although the equivalent pore network model simplifies the morphology and structure of the pores, it can better reflect the actual flow characteristics. The simulated permeability calculated based on the equivalent pore network model is basically the same order of magnitude as that based on actual test measurements^11,13,14^. Jerauld and Salter^16^ in 1990 compared the equivalent three-dimensional pore network model with the pore reconstruction model and found that the permeability calculation results of the two are equivalent. These validations enabled the equivalent pore network model to be considered as an established mathematical method to construct the pore structure of different rock and soil media based on flexibly based statistical pore parameters. However, unconventional shale geological conditions are very different; the solid phase usually consists of various mineral components in an intricate and anisotropic micro/nano pore structure^17,18^. Many shale gas formations have features such as heterogeneity, low matrix porosity, and low permeability, which is the critical factor that differentiates the unconventional formations from those conventional formations.

**Figure 1:**
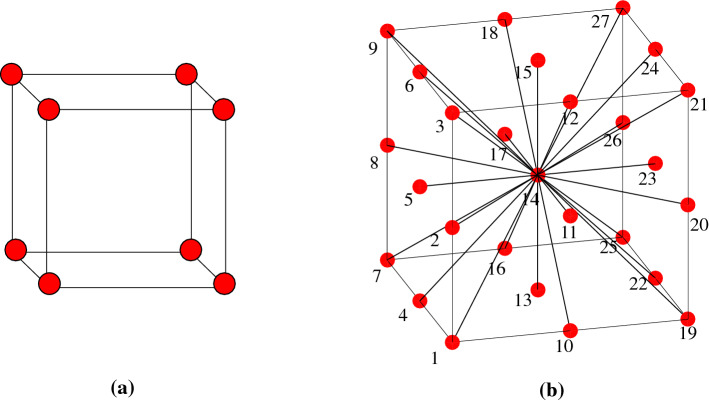
The geometry of the equivalent pore network. (**a**) Cubic lattice with a constant coordination number of six^15,20^. (**b**) Cubic lattices with 26 pore coordination number^21^.

An equivalent pore network is a simplified pore structure of the geo-media. It simplifies the complex flow paths (pore bodies, pore throats, etc.) in the geo-media into regular geometric shapes, such as spheres and cylinders, as shown in Fig. 1. This methodology gradually evolved from two-dimensional to three-dimensional and now developed into equivalent pore network models suitable for simulation of various geo-media. The pore bodies in an equivalent pore network represent the larger-sized cavities in the geo-media. The pores are connected by pore throats, smaller cavities, or nano-fractures for fluid migration. In an equivalent pore network, the size of pore radii varies, and they are regularly arranged on grid points. The distance between the centers of adjacent pores is a constant value to simplify calculations. The average number of surrounding pores varies depending on the geomaterial. The average number of active connections to a pore body is called the coordination number. By adjusting the size of the pore radius, pore throat radius and coordination number, a pore network model can be developed to simulate the gas seepage within geo-media and conventional reservoir rocks^19^.

Zhang et al.^22^ developed a nano-scale pore network model to describe the isotropic pore structure in shale formations based on a reduction factor for pore connections. This reduction factor depends on a dilution or a reduction algorithm. In this research, an anisotropic pore network model with an anisotropic ratio in three directions is developed by enhancing the model proposed by Zhang et al.^22^. This model is subsequently validated using measured permeability data.

### Motivation and importance of the research

The use of pore network models to study the seepage characteristics of rock and soil media has been carried out to a large extent, but there are still certain shortcomings. For its pore structure extraction and the equivalent anisotropic pore network still needs further development. Based on the equivalent pore network model, the relationship between the pore structure of shale and its seepage characteristics, especially anisotropic behavior, needs to be established. Using pore body sizes, pore throat sizes, coordination numbers, and other pore structure parameters, this manuscript presents the development of an equivalent anisotropic pore network model. This equivalent pore network can represent the average actual pore structure of shale so that the anisotropic permeability can be simulated. Geological media behaving as underground reservoirs generally have extremely low pore connectivities^20^. Macro scale gas flow simulations of such geological media usually ignore the impact of micro/nano-scale pore connectivity and flow regimes. The tight porosity and the complex connectivity of unconventional reservoirs make it difficult to account for the traditional macro/nano scale fluid flow. The establishment of a pore network model that can account for complex geometric shapes and spatial connectivity of pores and provide an effective means to explain the fluid flow in tight formations such as shales with micro/nano pores.

The method of establishing an equivalent pore network model has dramatically improved calculation efficiency^23^. The model can also account for the heterogeneous characteristics of the shale. Still, the results of the equivalent pore network model often deviate from the actual pore network, and further research on the model construction method is needed. Multi-scale flow characteristics will have a significant impact in the case of low permeability. Many studies have paid attention to the flow regime transition at different scales and pressures. Therefore, considering the multi-scale flow characteristics, the pore network model can predict the permeability of geotechnical materials with low permeability and connectivity and obtained results that are more consistent with measured test results.

### Parameters of the proposed pore network

The method for creating an equivalent pore network model with variable coordination numbers that can accommodate different pore-sizes, pore-throats, pore connections, and porosities, with a variable coordination number, is developed with the mathematical method of describing the pore network of shale in combination with data from the actual pore structure.

Via a regular three-dimensional lattice of pores connected by throats, the network model represents the void space of the rock. Each pore throat pore or pore body is presumed to be either cylindrical or spherical in a regularly spaced grid^16,24^. This assumption is the primary basis to develop a predictive model to characterizing the porous network of actual geomeaterials^25^. This model simplifies the complex seepage channels (pores, throats) geomaterials into regular geometric shapes, such as spheres, cylinders, etc., and arranges them regularly in the grid of a lattice. To construct the equivalent pore network as described above, the following six key model parameters are required:

- Pore body radius (*R*_p_) and its distribution: the pore body radius represents the size of the pores that are large cavities throughout the geological media.
- Pore throat radius (*R*_th_) and its distribution: the pore throat radius represents the size of the seepage channel between the pore bodies. Since any migration of fluid between the pore bodies must flow through pore throats, the pore throat size directly affects the seepage characteristics of the entire geological media.
- Coordination number (*ζ*) and its distribution: the pore coordination number represents the connectivity between pore bodies. For a geological media with high permeability, such as sand, one pore may be connected to multiple surrounding pores, and hence the coordination number is high (> 6). For a geological media with low permeability, such as shale, the pore coordination number is relatively small (< 4).
- Porosity (*n*): the porosity represents the proportion of the void in the geological media. Here, the voids include all the pore bodies and pore throats, including dead pore bodies and corresponding pore throats.
- Characteristic length (*L*): *L* is a concept introduced in an equivalent pore network representing the length of the mesh, which is the distance between adjacent pore bodies. L cannot be smaller than the average pore size of the two connecting pores.
- Anisotropy parameter: due to the different depositional factors of geo-materials, their hydraulic properties in different directions are different, which results in anisotropic permeability. The anisotropic parameter attempts to capture this hydraulic anisotropy.

The porosity is the ratio of the void volume to the total volume in the geological media. Here, the void volume includes the pore volume and the throat volume. Considering a single pore unit, the volume occupied by the pore body is:

1 $${V}_{\mathrm{p}}=\frac{4}{3}\times \pi \times {R}_{\mathrm{p}}^{3}$$

**Figure 2:**
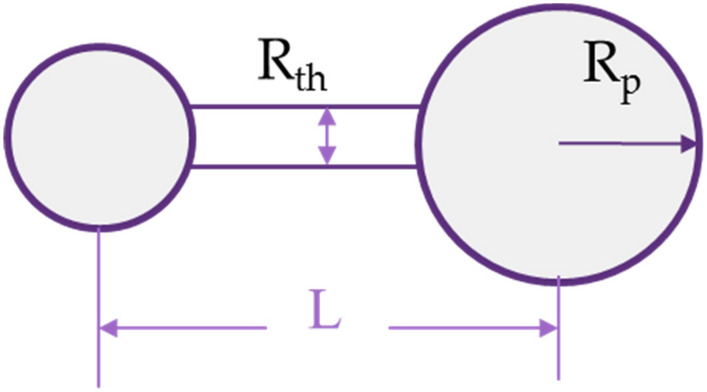
Pore connection in the pore network model.

The pore throat is assumed as a cylinder in the model (see Fig. 2), so its volume is determined by its throat radius and throat length. It should be noted that the length of the throat should be the distance between the pore centers, and the volume of pore throat function should be as follow:

2 $${V}_{\mathrm{th}}=\frac{}{2}\times \pi \times {r}_{\mathrm{th}}^{2}\times (L-2{R}_{\mathrm{p}})$$

where *ζ* is the coordination number, *R*_th_ is throat radius, and *L* is the unit size. Therefore, porosity can be expressed as:

3 $$n=\frac{{V}_{\mathrm{p}}+{V}_{\mathrm{th}}}{{L}^{3}}=(\frac{4\pi {R}_{\mathrm{p}}^{3}}{3}+\pi {R}_{\mathrm{th}}^{2}\times \left(\frac{L}{2}-{R}_{\mathrm{p}}\right)\times )/{L}^{3}$$

From the above formula, the relationship between porosity, pore radius, throat radius, pore coordination number, and unit length is established. Among the five parameters, the pore size *R*_p_, the throat size *R*_th_, the pore coordination number *ζ*, and the porosity *n* can be obtained directly from the experiments. Therefore, with the above parameters, one can construct the equivalent pore network.

### Construction of the anisotropic pore network

When the average coordination number in the equivalent pore network model is low, then there may be cases where the coordination number of some pores may be less than 2, and hence becomes dead-end pores (*ζ* = 1) or isolated pores (*ζ* = 0). There may also be dead-end pore groups and isolated pore groups (multiple pores have less than two connections to the main percolation channels). These pores are in the actual pore structure, but they do not contribute to the flow, so in the equivalent pore network model, they can be eliminated, thereby reducing computation time. In order to eliminate the pores that are not connected in the model, it is necessary to first define the isolated pores and the dead-end pores as follows:

1. Starting from any upstream pores, the passage is connected to any downstream pores through a non-repeating pore, called a seepage channel.
2. If a pore is contained by any seepage channel, it is called a seepage pore; otherwise, it is called a non-seepage pore.
3. For an interconnected non-seepage pore group, if there is a pore that can be connected to any seepage channel, this pore group is defined as a dead-end pore group. Otherwise, it becomes an isolated pore group.

**Figure 3:**
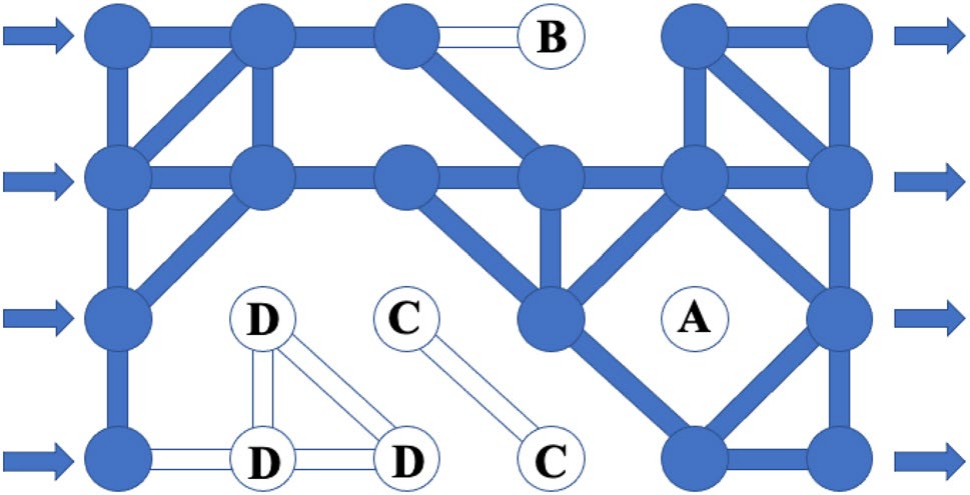
Schematic of pore types in the pore network.

According to the above definition, it can be seen that in the equivalent pore network shown in Fig. 3, pore A is isolated, pore B is dead-end pore, pores C belong to the isolated pore group, and pores D belong to the dead-end pore group. The dark region shows in Fig. 3 shows the seepage pores and pore throats after the pretreatment of the pore structure.

Through the above discussion, a generation method for the pore network model can be summarized as:

1. Pore radii generation: based on the distribution of pore body sizes, corresponding pore radii are randomly generated for each pore body in the network.
2. Pore connections: randomly assign a target coordination number to each pore body in the network and randomly generate inter-pore connections between all adjacent pores.
3. Using the elimination method to exclude isolated and dead-end pores in the network.

**Figure 4:**
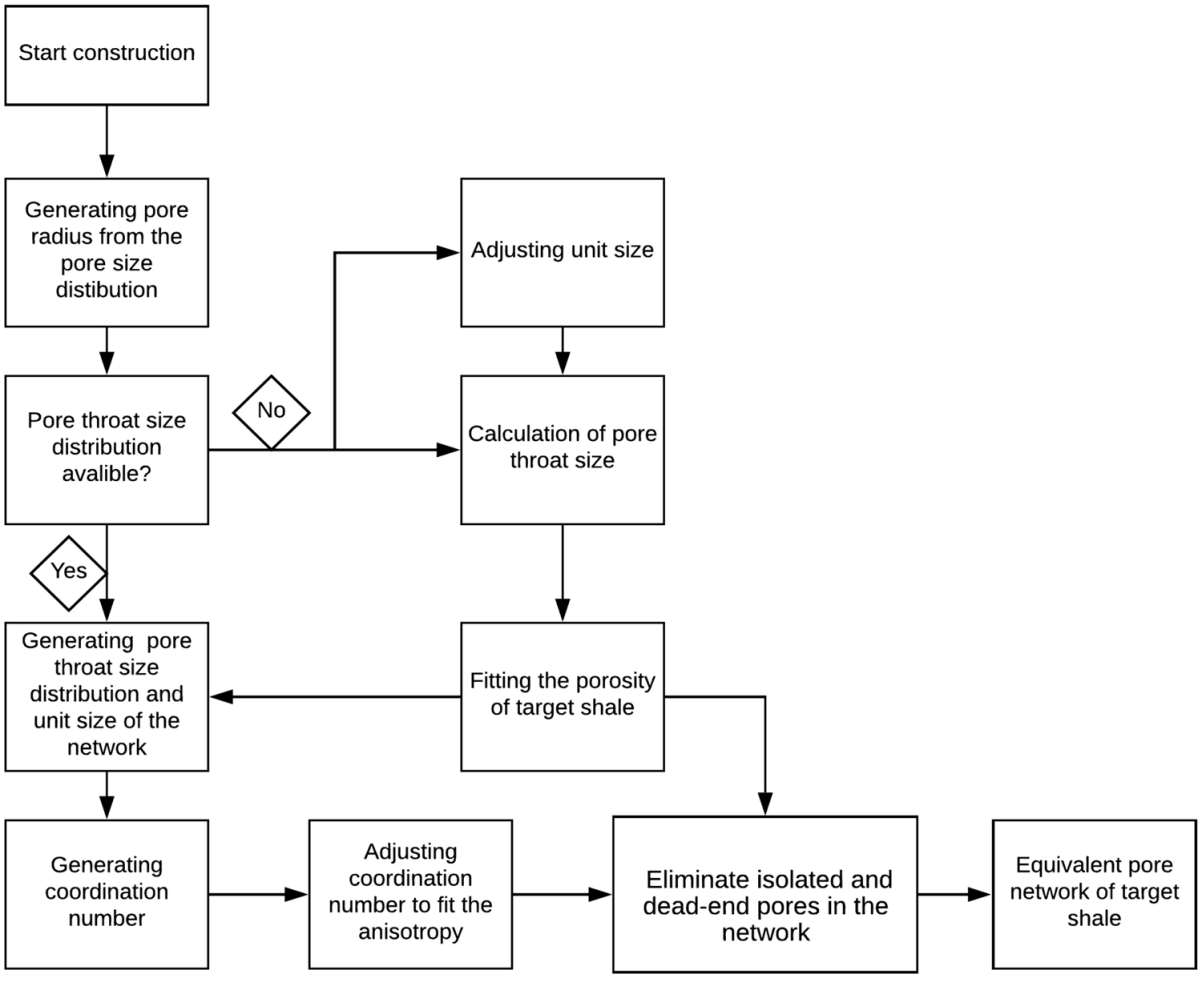
Calculation flow chart.

The flow chart of construction is shown in Fig. 4.

### Probability-based anisotropy and coordination number generation

**Figure 5:**
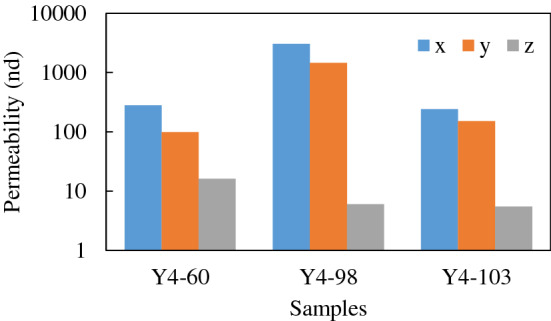
The anisotropic permeability of shale^31^.

Because shale is extremely anisotropic due to deposition and bedding, often the permeability in the vertical direction is lower when compared to that in the horizontal direction or the bedding plane^26^. The published permeability values vary by several orders of magnitude and are directly related to the applied effective stress (the difference between confining pressure and pore pressure) and bedding orientation relative to the flow direction (parallel or normal to bedding). Testing results of Mississippian Barnett Shale^27–29^ showed that permeability values ranged from 10^−17^ to 10^−21^ m^2^. For Scandinavian Alum and Toarcian Shales, Ghanizadeh, et al.^26^ reported permeability between 10^−17^ and 10^−22^ m^2^. In Western Canada for Woodford Shales, Pathi^4^ reported three to four orders of magnitude difference in anisotropic permeability. Tinni et al.^30^, found up to 100 times anisotropy for Devonian and Ordovician shale samples. Figure 5 shows the variation of permeability in different directions. This anisotropic behavior is incorporated into the pore network described in this research.

Figure 5 presents the average anisotropic permeability of three planes^31^. Under normal circumstances, it is unlikely to connect all the pores. Therefore, by setting different coordination numbers of the model in each direction, the anisotropy of geological media can be accounted. As for coordination number generation, the general practice was to delete the pore connections using a dilute algorithm after generating a fully connected structure^19^, or using a reduction factor proposed by Zhang et al.^22^, so that the pore connection is consistent with the real shale. The sparse algorithm described above can generate pore connections, but the following problems still exist: (1) the reduction factor is not obtained from the pore structure of the real geotechnical medium but selected and hence has certain subjective randomness; and (2) the sparse algorithm generally only satisfies the requirement of the average pore coordination number because it has a fixed deletion order but cannot support the coordination number distribution.

To account for anisotropy, one should analyze the randomly generated isotropic equivalent pore network. All connections between pores have the same probability, resulting in an equivalent pore network with an average pore coordination number of *E* = 26*p*, where *p* is the average probability of connection between the pores.

A simple analysis reveals that although the pore connections are randomly and uniformly distributed throughout the model, for pores with different pore coordination numbers, the probability of their connections to the surrounding pores is different^32^. For example, for one pore with 26 coordination numbers, the probability of connection will be 100%^33,34^, since the maximum connection number of a single pore of an equivalent pore network is 26. Here it is assumed that the probability of a connection between the pore bodies is only related to the coordination number of the two adjacent pores. It is essential to analyze the probability of connections of two pores within the pore network since this relationship will help to build the anisotropic relationship in three directions.

**Figure 6:**
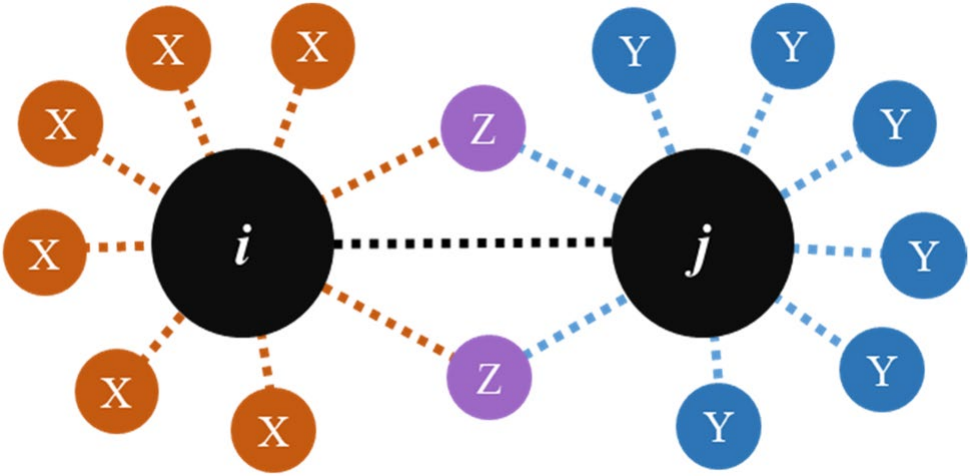
Connection relationship between pore i and pore j.

For convenience, the two adjacent pores may be referred to as pore *i* and pore *j*, respectively. It is then assumed that pore *i* is connected to pore *j* as shown in Fig. 6. Below we calculate the probability of a case where the coordination number of the pore *i* is *ζ*_i_, the coordination number of the pore *j* is *ζ*_j_.

For the pore *i*, the probability of generating *ζ*_i_ connections is *p*_1_:

4 $${p}_{1}\left({\zeta }_{\mathrm{i}}\right)=B({\zeta }_{\mathrm{i}};26,{p}_{\mathrm{c}})$$

where p_c_ represents the average probability of the existence of the connection between pores, and *B* represents the probability distribution of the binomial distribution *B*(*k*; *n*, *p*), where it is defined as:

5 $$B\left(k;n,p\right)=\left(\begin{array}{c}n\\ k\end{array}\right){p}^{k}{\left(1-p\right)}^{n-k}$$

In the generated pore network, the probability that the pores i and the pores j are connected is expressed as:

6 $${p}_{2}\left(x\right)={p}_{1}\left({\zeta }_{\mathrm{i}}\right)\cdot \frac{{\zeta }_{\mathrm{i}}}{26}=B\left({\zeta }_{\mathrm{i}};26,{p}_{\mathrm{c}}\right)\cdot \frac{{\zeta }_{\mathrm{i}}}{26}$$

Since the pores *i* has been connected to the pores *j*, it is possible to randomly generate pores in the remaining 25 connections, whereby the probability of forming a connection between the two is *p*_3_:

7 $${p}_{3}\left({\zeta }_{\mathrm{i}},{\zeta }_{\mathrm{j}}\right)={p}_{2}\left({\zeta }_{\mathrm{i}}\right)\cdot B\left({\zeta }_{\mathrm{j}}-1;25,{p}_{\mathrm{c}}\right)=B\left({\zeta }_{\mathrm{i}};26,{p}_{\mathrm{c}}\right)\cdot \frac{{\zeta }_{\mathrm{i}}}{26}\cdot B\left({\zeta }_{\mathrm{j}}-1;25,{p}_{\mathrm{c}}\right)$$

Equation (7) shows the probability of a connection between two pores. Similarly, the probability that there is no connection between the two pores can also be calculated. Therefore, the probability that no connection occurs between the two pores is $\stackrel{-}{{p}_{3}}$:

8 $$\stackrel{-}{{p}_{3}}\left({\zeta }_{\mathrm{i}},{\zeta }_{\mathrm{j}}\right)=B({\zeta }_{\mathrm{i}};26,{p}_{\mathrm{c}})\cdot (1-\frac{{\zeta }_{\mathrm{i}}}{26})\cdot B\left({\zeta }_{\mathrm{j}};25,{p}_{\mathrm{c}}\right)$$

In summary, given the two coordination numbers of these adjacent pores, the conditional probability of the connection between the pores is:

9 $$p\left({\zeta }_{\mathrm{i}},{\zeta }_{\mathrm{j}}\right)=\frac{{p}_{3}\left({\zeta }_{\mathrm{i}},{\zeta }_{\mathrm{j}}\right)}{{p}_{3}\left({\zeta }_{\mathrm{i}},{\zeta }_{\mathrm{j}}\right)+\stackrel{-}{{p}_{3}}\left({\zeta }_{\mathrm{i}},{\zeta }_{\mathrm{j}}\right)}=\frac{\stackrel{-}{{\zeta }_{\mathrm{i}}}\stackrel{-}{{\zeta }_{\mathrm{j}}}}{{p}_{\mathrm{c}}}/(\frac{\stackrel{-}{{\zeta }_{\mathrm{i}}}\stackrel{-}{{\zeta }_{\mathrm{j}}}}{{p}_{\mathrm{c}}}+\frac{(1-\stackrel{-}{{\zeta }_{\mathrm{i}}})(1-\stackrel{-}{{\zeta }_{\mathrm{j}}})}{1-{p}_{\mathrm{c}}})$$

Among them $\overline{{\zeta_{i} }} = \zeta_{i} /26$, $\overline{{\zeta_{j} }} = \zeta_{j} /26$ respectively, are called normalized coordination numbers of two pores.

**Figure 7:**
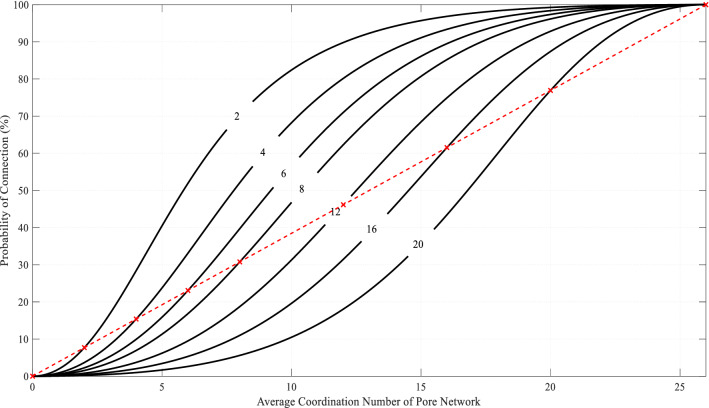
Relationship between the probability of connections and the average coordination number.

It can be seen from Eq. (9) that the probability of connection between pores is not only related to the coordination number of the two pores but also the average coordination number of the entire equivalent pore network model. Figure 7 shows the relationship between the probability of a connection between pores and the average coordination number of the network when the coordination number of the two pores is the same. Results show that when the average coordination number of the network is constant, the larger the pore coordination number, the greater the probability of connection between the pores. When the average coordination number of connected pores is constant, the larger the average coordination number of the network, the larger the probability of connection between the pores. The points on the dash straight line in Fig. 7 shows that the pore coordination number on each curve is equal to the average coordination number of the network, indicating that when the coordination number of the two pores is the same as the average coordination number of the network.

Based on the above, one can use the known coordination number distribution to generate the connection between the pores, using the following steps:

1. Randomly generate a series of matches according to a given distribution of coordination numbers and assign target coordination numbers to each pore. In the process of coordination number assignment, a larger coordination number is assigned to the larger size pores to meet the actual statistical results. In our case, the coordination number has a general deviation of 0.1, which is relatively small.
2. For any two adjacent pores, based on the target coordination number assigned to the two pores, combined with the average coordination number of the entire model, the probability of the connection between the groups of pores is calculated by using Eq. (9).
3. Make adjustments to all pores until the final pore network is generated.

**Figure 8:**
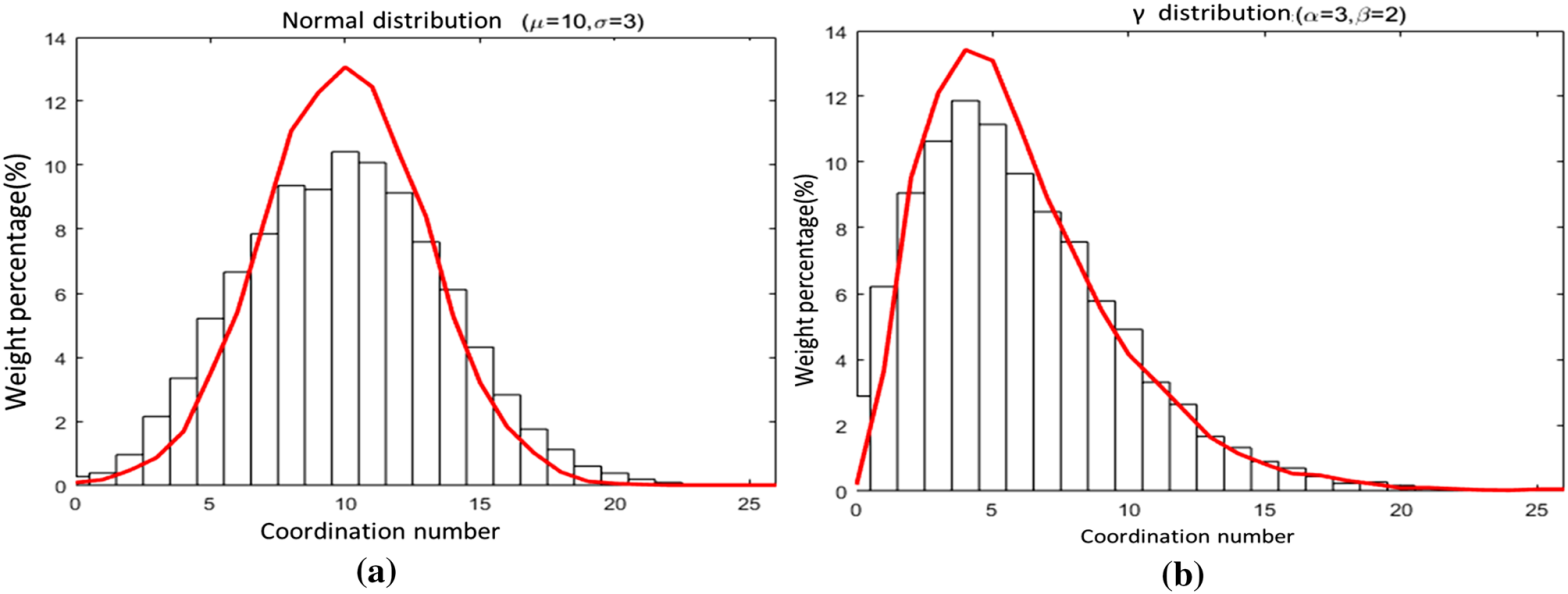
Equivalent pore network model connection generation results (**a**) normal distribution (**b**) gamma distribution.

The equivalent pore network model generated here avoids the strong subjective reduction factor. The generated connection considers the distribution of coordination numbers. It can generate an equivalent pore network that is similar to actual geo-material. Figure 8 and Table 1 show the connection of the equivalent pore network model with the target coordination number and the normal distribution and the gamma distribution using the above method where *μ* and *α* are the average numbers of normal distribution and gamma distribution, and *σ* and *β* are the standard deviations of both distributions, respectively. The distribution of the coordination number and the target coordination number are close with a mean error is less than 1%. However, gamma distribution has a lower error, and hence it was used in this research.

**Table 1:** Comparison between two distributions.

Target distribution	Average coordination number			Standard deviation		
	Target	Actual	Error (%)	Target	Actual	Error (%)
Normal distribution(*μ* = 10, *σ* = 3)	9.99	9.97	− 0.14	3.01	3.74	+ 24.23
γ distribution(*α* = 3, *β* = 2)	5.96	5.92	− 0.82	3.43	3.74	+ 8.91

**Figure 9:**
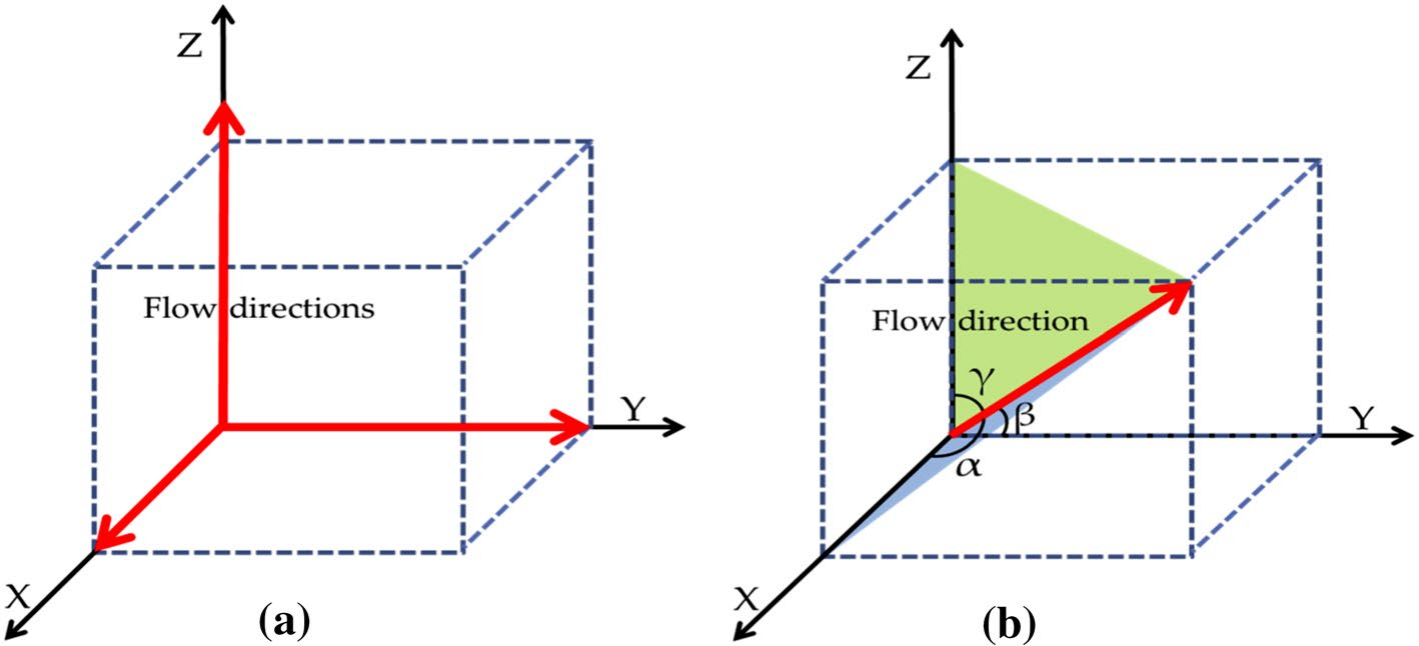
Space diagonal connection and angles schematic (**a**) flow in *x*, *y* and *z* directions (**b**) flow in space.

Actual shale formations are anisotropic, and different directions often exhibit different permeabilities. In the process of model building, an anisotropic parameter can be used in the equivalent pore network with different connection probabilities. Since the developed pore network model is three-dimensional, a new concept was introduced to consider the anisotropic permeabilities for three different planes of shale matrix: anisotropic ratio. It is defined as the ratio of the number of pore connections in the three directions of *x*, *y*, and *z* as (*a*_x_, *a*_y_, *a*_z_); since the developed model is a regular lattice grid, the total number of connections for each plane is available after the construction also for the angels between connections to *x*, *y,* and *z*-axis, in this case (regular lattice cubic grid), angles between the space diagonal to each of the axis within a cubic would be 54.7° defined as *α*, *β* and *γ*, respectively shown as Fig. 9. When the certain shale media of given anisotropy ratio, the number of pore connections in the three directions of *x*, *y* and *z* are first guaranteed. To calculate the probability in other directions (*α*, *β* and *γ* directions) other than *x*, *y* and *z* directions, Eq. (10) can be used, and connections to this lattice model are determined based on this connection probability.

10 $$\frac{p\left(\alpha ,\beta ,\gamma \right)}{p\left({\zeta }_{i},{\zeta }_{j}\right)}=\frac{{a}_{x}{cos}^{2}\alpha +{a}_{y}{cos}^{2}\beta +{a}_{z}{cos}^{2}\gamma }{\stackrel{-}{a}}$$

where *α*, *β*, and *γ* are the angles between the connection direction and the *x*, *y*, and *z*-axis as shown in Fig. 9b, whereas Fig. 9a shows traditional flow directions in direction and the *x*, *y*, and *z*-axis. The $\stackrel{-}{a}$ is defined as the average connections of the whole pore network. This equation describes the relationship between pore connection probability within a lattice grid and their spatial angles to *x*, *y* and *z* directions. When the lattice grid was constructed, the pore connection could be calculated based on this theory. As for the angles of *α*, *β* and *γ* in the equation, they can be calculated based on the basic lattice unit, as in this case: a cube with the same height, length, and width. In other cases, when the basic lattice unit is cuboid with different lengths, widths, and heights, this equation can also be utilized to account for a more complicated connection.

The anisotropic equivalent pore network obtained by this method has a similar total number of connections to the isotropic network. However, the seepage behaviors of the three planes are different, which is validated by the anisotropic permeability experiments for shales.

## Calculation of gas permeability

### Gas movement in pore throats and a pore

In the pore network, the gas transfer through the pore throats connected between pores. The gas flux of the pore throat *Q*_ij_ can be determined as follows:

11 $${Q}_{\mathrm{ij}}={\alpha }_{\mathrm{ij}}{K}_{\mathrm{ij}}\Delta p$$

where *α*_ij_ is a correction factor, *K*_ij_ is the liquid permeability of the pore throat, *Δp* is the pressure difference between the pores.

The correction factor *α*_ij_ can be determined by the multi-flow regimes model provided by Zhang et al.^35^, which includes the influence of slippage effect and Knudsen diffusion of gas in shale:

12 $${\alpha }_{\mathrm{ij}}=\frac{1+4{K}_{\mathrm{n}}+\frac{32}{3\pi }{{K}_{\mathrm{n}}}^{2}}{1+0.5{K}_{\mathrm{n}}}$$

where *K*_n_ is the Knudsen number ^36^, which can be expressed as:

13 $${K}_{\mathrm{n}}=\sqrt{\frac{\pi RT}{2M}}\frac{\mu }{p}$$

where *R* is the universal gas constant, *T* is the absolute temperature, *M* is the molar mass, *μ* is the viscosity of the gas, *p* is the average gas pressure in the pore throat.

The *K*_ij_ can be determined by Hagen–Poseuille equation^37^:

14 $${K}_{\mathrm{ij}}=\frac{\pi {{r}_{\mathrm{ij}}}^{2}}{8\mu {l}_{\mathrm{ij}}}$$

where *r*_ij_ and *l*_ij_ are the radius and the length of the pore throat.

Due to the compressibility of the gas, the mass balance of a pore connected to several pore throats can be written as:

15 $${Q}_{\mathrm{pore}}=\sum \frac{2{p}_{\mathrm{i}}}{{p}_{\mathrm{i}}+{p}_{\mathrm{j}}}{Q}_{\mathrm{ij}}$$

where *Q*_pore_ is the flux of the pore, *p*_i_ and *p*_j_ are the pressure at both ends of the pore throat. Based on Eqs. (11–15), the gas transfer in the pore network can be solved. When *α*_ij_ is set as 1, the equations can be used to calculate liquid transport in pore throats.

### Macro permeability of the pore network

Considering one-dimensional seepage with constant pressure at the upstream and downstream boundaries, when the gas flow form a stable field and the density of the gas gradually changes with the pressure.

16 $$\frac{\mathrm{d}p}{\mathrm{d}L}=\frac{\mu }{\rho }\frac{{Q}_{\mathrm{m}}}{kA}=\frac{\mu }{p}\frac{RT}{M}\frac{{Q}_{\mathrm{m}}}{kA}$$

where *Q*_m_ is the total mass flux of gas in the model, *ρ* is the density of the fluid, *k* is the macro gas permeability of the medium, *A* is the seepage area. After integration, Eq. (16) changes into:

17 $${p}_{\mathrm{u}}^{2}-{p}_{\mathrm{d}}^{2}=\frac{\mu RT}{M}\frac{{Q}_{\mathrm{m}}}{kA}L$$

where *p*_u_ and *p*_d_ are the constant pressure at the upstream and downstream boundaries of the model, *L* is the seepage distance of the model. The macro gas permeability of the entire equivalent pore network model can be calculated as shown below:

18 $$k=\frac{2\mu RT}{{(p}_{\mathrm{u}}+{p}_{\mathrm{d}})M}\frac{L}{A}\frac{{Q}_{\mathrm{m}}}{{p}_{\mathrm{u}}-{p}_{\mathrm{d}}}=\frac{\mu }{\stackrel{-}{\rho }}\frac{L}{A}\frac{{Q}_{\mathrm{m}}}{{p}_{\mathrm{u}}-{p}_{\mathrm{d}}}$$

where $\stackrel{-}{\rho }$ represents the average density of gas in the entire pore network. Please note that Eq. (18) can also be used to calculate the macro permeability of liquids by replacing average gas density with average liquid density in the pore network.

## Results and validation

### Validation of the anisotropic model

The experimental results show that the permeability of Longmaxi formation shale in East Sichuan is in the order of 10^–18^ (in m^2^)^38^. With the increase of the angle between the shale and the bedding plane, the permeability of shale decreases. The anisotropic permeability ratio of the Longmaxi shale along the bedding plane and the permeability of shale sampled on the vertical bedding plane varies from 3 to 10^38^. The permeability values of the shale samples in a different area of the bedding plane are relatively similar, but the permeability of different rock samples varies with the confining stress. As for the anisotropic pore network used in this research, the reasonable anisotropic ratio has to be adopted based on the previous research and the relationship between the anisotropic ratio, which refers to the connection number ratio in each plane as (*a*_x_:*a*_y_:*a*_z_).

**Figure 10:**
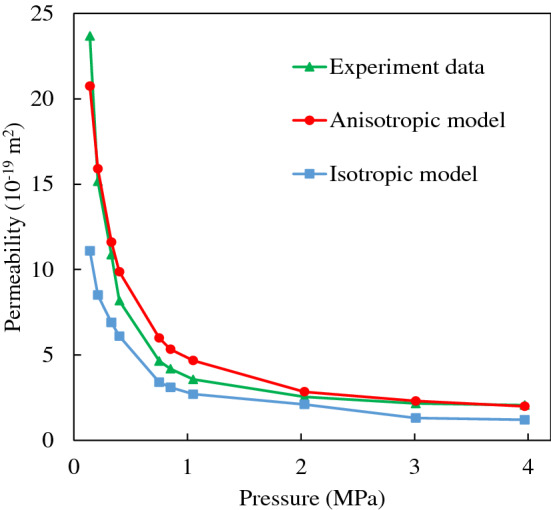
Comparison of permeability from experimental data and isotropic/anisotropic pore-network model (experiment data is Sample Z084, isotropic data and anisotropic data was compared).

Based on the statistical study of this anisotropic pore network model, the initial results between the anisotropic permeability and the connection ratio for three flow planes, as shown in Table 2. The following are assumed for the calculations shown in Table 2: average pore body radius 0.1 μm and standard deviation 0.01; average coordination number 10 and standard deviation 0.1; average pore throat radius 0.1 μm and standard deviation 0.01; and porosity 10%. This table indicates that anisotropic permeability can be achieved using this connection ratio of three planes. Each of the factors has a different impact on permeability. The dominating flow plane (*a*_x_), which is the bedding plane, has a significant impact on permeability. When increasing the ratio of *a*_x_, the total permeability tends to increase, which means a higher connection number for the bedding plane will increase the permeability of the whole matrix. On the contrary, the increase in the ratio of *a*_y_ and *a*_z_ will decrease the total permeability. It is reasonable that when increasing the vertical connections of shale, the permeability of the total formation will decrease. The isotropic pore network model was unable to predict the shale permeability when the pressure difference was low as shown in Fig. 10. Whereas the anisotropic model seems to predict the permeability for a wide range of input pressure values. Typically, when the pressure ranges from 0.1 to 1 MPa, the isotropic model tends to predict a lower permeability value when compared to the measured values. On the contrary, the permeability results of an anisotropic model, when adjusted to include the proper connection ratio, yielded similar values to those measured. Therefore, by applying and adjusting the connections ratio of each plane, one can use the anisotropic pore network model to represent the real shale formation. For Longmaxi shale, the typical anisotropic ratio is approximately 10.

**Table 2:** Impact of connection ratio on permeability.

(*a*_x_:*a*_y_:*a*_z_)	1, 1, 1	1, 2, 1	1, 5, 1	1, 10, 1	1, 50, 1	1, 100, 1
Permeability (m^2^) (X-direction)	4.11 × 10^–18^	3.72 × 10^–18^	3.32 × 10^–18^	3.11 × 10^–18^	2.89 × 10^–18^	2.84 × 10^–18^
(*a*_x_:*a*_y_:*a*_z_)	1, 1, 1	2, 1, 1	5, 1, 1	10, 1, 1	50, 1, 1	100, 1, 1
Permeability (m^2^) (X-direction)	4.11 × 10^–18^	5.94 × 10^–18^	8.13 × 10^–18^	8.90 × 10^–18^	9.28 × 10^–18^	9.36 × 10^–18^
(*a*_x_:*a*_y_:*a*_z_)	1, 1, 1	1, 1, 2	1, 1, 5	1, 1, 10	1, 1, 50	1, 1, 100
Permeability (m^2^) (X-direction)	4.11 × 10^–18^	3.75 × 10^–18^	3.38 × 10^–18^	3.12 × 10^–18^	2.90 × 10^–18^	2.86 × 10^–18^

### Validation of the model with experimental data from Qaidam Basin Shale

**Figure 11:**
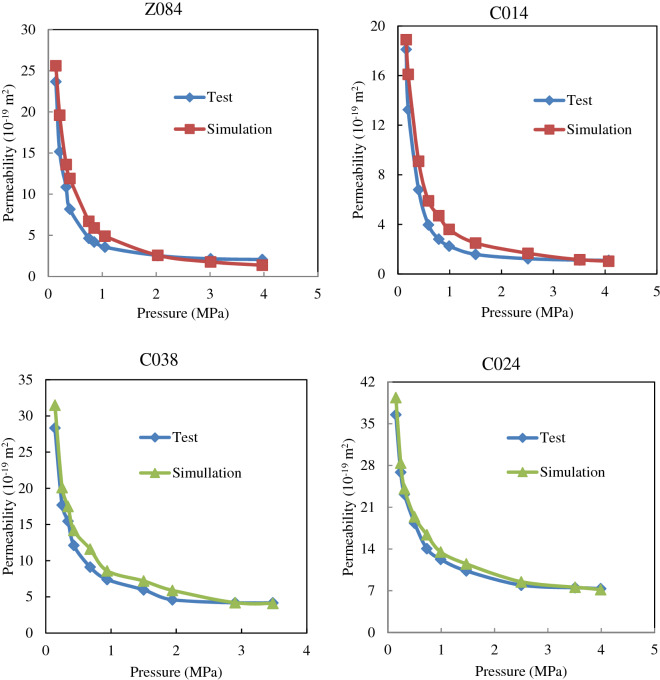
Variation of the permeability with applied pressure for the four formations with the simulated results.

Gao et al.^39,40^ conducted a series of high-pressure gas permeability tests on four shale samples obtained from Longmaxi formation shale in East Sichuan. Several different types of shale samples were used for the test, and the representative sample parameters are shown in Table 3. For each sample, multiple sets of permeability tests under different pressures were performed. The outlet pressure of the test was fixed at 0.1 MPa, and the inlet pressure was gradually increased from 4 to 0.1 MPa. By measuring the corresponding volumetric flow rate, the apparent permeability of the shale medium at the corresponding pressure was calculated, as shown in Fig. 11.

**Table 3:** Shale sample parameters ^22,39,41,42^.

Sample	Pore diameter /standard deviation (nm)	Pore throat diameter/standard deviation (nm)	Coordination number/standard deviation	Anisotropic ratio (*a*_x_:*a*_y_;*a*_z_)	Porosity (%)
C024	5.187, 0.4	0.51, 0.04	4, 0.3	17:17:1	5.98
C038	7.852, 0.4	0.81, 0.04	4, 0.3	22:22:1	5.34
C014	7.305, 0.4	0.87, 0.04	3, 0.3	25:25:1	2.55
Z084	4.333, 0.4	0.66, 0.04	3, 0.3	29:29:1	2.12

Pores of Longmaxi shale are composed of organic and non-organic pores in the shale fabric, and micro and macro cracks. These pores store shale gas, and their size directly determines the shale gas reservoir size and the feasibility of shale gas extraction. The anisotropic pore network was constructed based on the reported pore parameters of four different shales and shown in Table 3. Mercury injection capillary pressure test, N_2_ absorption, and FIB-SEM technologies were used to obtain pore parameters of four Longmaxi shales under laboratory conditions^40^.

The low-pressure absorption test supports the assumption of equivalent spherical and cylindrical pore shapes^36^. To simplify the understanding of the pore structure and to facilitate the prediction of gas flow, the pore structure in the shale matrix was simplified to cylindrical capillaries^37–39^. Hence the shale matrix pores consist of a large number of multi-diameter cylindrical pores connected spherical pore bodies in series and parallel.

Four anisotropic pore networks were developed for four shale samples obtained from the Longmaxi formation shale in East Sichuan using the steps described before. The size of each anisotropic pore network was 20 × 20 × 20. The pore diameters and porosity values were obtained from the experimental data reported in Gao et al.^34,35^. In that study, they conducted different tests including CO_2_ absorption, N2 adsorption and mercury intrusion to obtain pore structures of four Longmaxi shales under laboratory conditions. Other parameters including the pore throats diameters, coordination numbers and anisotropic ratios, were based on our previous and current research on shale^19,40–42^.

Then using those four anisotropic pore networks developed the permeability of each shale was predicted. No flow boundary conditions were used, except for the inlet or upstream and outlet or downstream boundaries. By setting the outlet pressure to 0.1 MPa, one can simulate the results of gas flow by changing the values of the inlet pressure and calculate the corresponding permeability. For each core sample, several simulations were performed corresponding to different inlet pressures of actual physical tests. Once the inlet and outlet pressures were applied, the simulation continued until a constant flow rate was reached.

Based on the summary of the modeling parameters shown in Table 3, four permeability results of both actual test and simulation were compared in Fig. 11. These simulations used the developed anisotropic pore network. Figure 11 shows the comparison between the experimental results and the results of the equivalent pore network model simulation. It can be seen from Fig. 11 that the simulation results of the pore-scale permeability calculation model are in good agreement, concluding that the proposed equivalent anisotropic pore network model can be used as a predictive tool.

For unconventional rock such as shale, porosity is usually less than 10%^48^, pore-throat diameters are less than 1 μm^49^, and permeability is less than 1 mD ^43–45,51^. Shale formations are strongly anisotropic due to their laminated surfaces, resulting in vast differences in mechanical properties along parallel and perpendicular lines to bedding planes^46,47,50^. Also, shale is highly anisotropic when compared to conventional rock. Shale nano-pores are heterogeneous and contain organic (e.g., kerogen) and inorganic (e.g., clay and cementation) materials^52^. Hence at nano/micro-scale flow simulations, the approach proposed by Song et al.^11^ with pore structure obtained by scanning-electron-microscopy (FIB-SEM) and then using the local-effective-viscosity multi-relaxation-time lattice Boltzmann model to simulate the gas transport in shale would be preferable.

However, the gas trapped in shale is influenced by conditions that prevailed during reservoir-formation and subsequent environmental and seismic activities^53^, which is a complex balancing operation, hence in the case of unconventional hydrocarbon resources, the gas content is affected by temperature and pressure in micro and nano scale-pores^35,44–47,54^. To better estimate the potential for gaseous hydrocarbon recovery, it is essential to characterize these heterogeneous nano-pores using pore-scale parameters, i.e., the pore body and throat distributions^22^. Hence at engineering scale for the prediction of potential shale gas extraction, the simplified pore structure of the geo-media using the equivalent pore network incorporating anisotropy proposed in this manuscript may be preferred.

## Summary and conclusions

This manuscript describes the development of an improved pore-network model using a new algorithm for coordination numbers incorporating anisotropic ratio as well as the isolated pore elimination method. The model was validated by comparing simulated shale permeability with the measured data for four shale from the Longmaxi formation. The construction of the equivalent anisotropic pore network model was first described in detail, especially the proposed coordination number generation method and the assignment of coordination numbers based on the connection probability of adjacent pores. The probability of connection was developed and found that the connection between two pores is not only related to the coordination number of two pores but also related to the average coordination number of the entire network. An anisotropic ratio (*a*_x_:*a*_y_:*a*_z_) was then introduced to account for the anisotropic formation of gas-bearing shale. To accurately represent the permeability behavior of different anisotropic formations such as soils, sandstones, or shale, the three directional anisotropic ratios were defined as the ratio of pore connections in each direction. For different formations, different anisotropic ratios were utilized to simulate different gas flow behaviors. A good agreement between predicted and measured permeability was obtained for four shale samples obtained from the Longmaxi formation in East Sichuan, showing that the anisotropic model is more representative than the conventional isotropic pore network model to represent pore connections in shale formations.

## Data Availability

No experiments were performed, and hence there are no experimental data or material.

## Code availability

We will share the computer code developed free of charge with any interested researchers.
